# SEARCHBreast: a new online resource to make surplus material from in vivo models of breast cancer visible and accessible to researchers

**DOI:** 10.1186/s13058-016-0716-2

**Published:** 2016-05-31

**Authors:** Bethny Morrissey, Karen Blyth, Phil Carter, Claude Chelala, Louise Jones, Ingunn Holen, Valerie Speirs

**Affiliations:** Leeds Institute of Cancer and Pathology, University of Leeds, Leeds, UK; Cancer Research UK Beatson Institute, Glasgow, UK; Barts Cancer Institute, London, UK; Academic Unit of Clinical Oncology, University of Sheffield, Sheffield, UK

Progress in understanding breast cancer biology is often underpinned by pre-clinical studies in mice. Upon completion of these studies, surplus tissues are frequently archived but often never used. There is a pressing need to make this material available so it can be shared with other research groups to answer additional scientific questions, saving time and cost by avoiding unnecessary animal models being created and, in many instances, duplicated. As well as increasing research outputs, this will reduce animal use, helping to address the 3Rs—Replacement, Refinement, and Reduction—in animal research [1].

We would like to draw attention to a new resource we have developed to help facilitate the collaborative sharing of archival tissues derived from mouse models of breast cancer [2]. Called SEARCHBreast (Sharing Experimental Animal Resources: Coordinating Holdings—Breast), this virtual resource enables researchers to access well-characterised excess animal materials reducing the need to initiate new in vivo studies.

The SEARCHBreast database (https://searchbreast.org) contains information on thousands of tissue samples available for immediate use following a simple online request. Samples are available from over 40 different mouse models, including transgenic, xenograft and patient-derived xenografts (PDXs). Available tissues include mammary tumours, normal mammary glands, mammary epithelial cells, and metastases (lung, liver, brain, bone). Information including genetically engineered mouse (GEM) alleles, background strain, cell lines, metastatic sites and penetrance, transplantation site, and hormone receptor status is also available. Importantly, these samples are ready for immediate use, with most available as paraffin embedded blocks or histological slides. Through bypassing the animal development pipeline which can take up to 2 years and cost upwards of US$140,000 [2], utilising the SEARCHBreast database can save scientists both time and money. This is particularly relevant for PDX models which are costly to generate and maintain but have a growing use in many areas of breast cancer research [3]. The material remains with the owner who is under no obligation to share but may be open to collaboration. SEARCHBreast is a no-cost mediator to facilitate these collaborations. Additionally, SEARCHBreast enables scientists with little experience of in vivo work the opportunity to perform experiments under the guidance of the material owner, which could increase the quality of their research, enhancing their research output and impact [2]. By registering at https://searchbreast.org, academic researchers have free access to this innovative resource.

## Abbreviations

PDX: patient-derived xenograft
SEARCHBreast: Sharing Experimental Animal Resources Coordinating Holdings—Breast
